# Evaluating the diagnostic value of multi-detector brain CT angiography in diagnosing acute cerebral venous thrombosis

**DOI:** 10.1038/s41598-022-21743-x

**Published:** 2022-11-04

**Authors:** Bita Abbasi, Nadia Kahani, AmirAli Moodi Ghalibaf, Parvaneh Layegh, Shabnam Niroumand, Reza Akhavan, Ehsan Hamidi, Maryam Salehi

**Affiliations:** 1 Department of Radiology, Faculty of Medicine, Mashhad University of Medical Sciences, Mashhad, Iran; 2 Student Research Committee, Faculty of Medicine, Birjand University of Medical Sciences, Birjand, Iran; 3 Department of Social Medicine, Faculty of Medicine, Mashhad University of Medical Sciences, Mashhad, Iran; 4 Department of Emergency Medicine, Faculty of Medicine, Mashhad University of Medical Sciences, Mashhad, Iran; 5 Department of Urology, Faculty of Medicine, Mashhad University of Medical Sciences, Mashhad, Iran

**Keywords:** Diagnosis, Medical imaging

## Abstract

Cerebral venous sinus thrombosis (CVST) is a rare type of venous thromboembolism mostly affecting young adults. Despite improved imaging studies, the diagnosis is usually delayed by several days. An average diagnostic delay of seven days from the onset of symptoms is still reported for this condition, and it is crucial for radiologists to detect this potentially lethal condition in routine imaging studies. In this study we aimed to investigate the diagnostic value of multi-detector brain CTA in diagnosing acute CVT. We searched our Picture Archiving and Communicating System (PACS) of our tertiary-level academic hospital between March 2016 and March 2019, and collected all patients for whom both contrast-enhanced MRV and brain CTA were acquired at the same admission. A total of 242 patients were found on our PACS database who met our criteria. In the blinded multidetector-row computed tomographic angiography (MDCTA) evaluation, there was a sensitivity of 96.1%, specificity of 98.6% and accuracy of 98.3% for MDCTA in detecting CVST. In the emergency settings, and in centers in which MRI scanners are not available, MDCTA can be used instead of CE-MRV for diagnosis of CSVT with a good sensitivity and specificity.

## Introduction

Cerebral venous sinus thrombosis (CVT) is a rare type of venous thromboembolism mostly affecting young adults^1^. It comprises 0.5–3 percent of stroke cases^2^. Patients can present with a diverse range of symptoms such as headache, visual disturbances, focal neurological deficits, etc.^3–7^. In contrast to arterial strokes, symptoms develop in a more insidious manner, making the diagnosis more difficult and highly dependent on clinical suspicion^8,9^. Although increased awareness of this condition and improved imaging studies have led to better prognosis, but the mean duration for this condition's diagnosis is seven days from the onset of symptoms^7,10^. CVT should always be considered as a differential diagnosis in young adults presenting with unusual headache or stroke-like symptoms.

The most sensitive noninvasive method for diagnosing CVT is contrast enhanced magnetic resonance venography (CE-MRV)^10,11^. However, MRI is a time-consuming modality and is not available in many healthcare centers, especially in the emergency settings. The need for expert radiologist judgment is another disadvantage of MRI^10,12^. Recently, multidetector-row CT angiography (CTA) is gaining popularity as a diagnostic method for CVT^13–16^. As the arterial and venous phase in the brain circulation are not far apart, cerebral venous sinuses are usually opacified in the CTA studies and can be fully evaluated. A few studies reported great sensitivity and specificity for this modality for diagnosis of CVT^17,18^, however, the number of studies are still limited. In this study we aimed to investigate the diagnostic value of multi-detector brain CTA in diagnosing acute CVT and compared it with contrast-enhanced MRV as the reference standard.

## Methods

This retrospective study was carried out according to relevant guidelines and regulations. The Ethics committee of Mashhad University of Medical Sciences (MUMS) approved this retrospective observational study. The review board at MUMS waived informed consent due to the observational nature of the research, under the waiver statement IR.MUMS.MEDICAL.REC.1398.470.

### Subjects

All methods were performed in accordance with the relevant guidelines and regulations.

To achieve the patients' imaging data, the Picture Archiving and Communicating System (PACS) of our tertiary-level academic hospital was searched between March 2016 and March 2019 and collected all patients for whom both contrast-enhanced MRV and brain CTA were acquired at the same admission. Inclusion criteria were patients with acute neurologic symptoms who were evaluated for CVT, age above eighteen years, available CE-MRV and a brain CTA obtained within seven days from MRV. The exclusion criteria were the presence of severe artifacts in MRV or CTA making the interpretation challenging and incomplete imaging protocol. A total of 245 adult patients were identified. The mean age of patients was 42.5 (+ 15.2) years. There were 104 (42.4%) male and 141 (57.6%) female patients.

### Imaging

All multidetector-row computed tomographic angiography (MDCTA) examinations were performed with a commercially available 16-MDCT scanner (Neusoft, Neuviz 16). All patients received 150 mL of contrast material (Visipaqu™ 320 mg/mL) with the flow rate of 4 mL/s, followed by 50 mL of saline chaser injected with the same flow rate.

### The reference standard imaging

Although the gold standard for diagnosing CVT is digital subtraction angiography, this technique is invasive and has been replaced with MRI + MRV in many centers^10^. We used a combination of MRI and contrast-enhanced MRV as the reference standard for diagnosing CVT.

MR imaging was performed on 1.5 T scanner (MagnetomVision; Avanto; I-class), including diffusion-weighted images (TR, 3500 ms; TE, 104 ms; section thickness, 5.5 mm; B-value, 1000), axial proton attenuation-and T2-weighted images, (TR, 4020 ms; TE 107 ms; section thickness, 5 mm), T1-weighted (TR, 388 ms; TE, 15 ms; section thickness, 5 mm), coronal fluid-attenuated inversion recovery (FLAIR) (TR, 9610 ms; TE, 90 ms; TI, 2564.5 ms; section thickness, 5 mm) sequence, and venous 2D time-of-flight (TOF) MR angiography (TR, 31 ms; TE, 7.86 ms; flip angle, 60°; section thickness, 3 mm) with superior arterial saturation pulses.

The contrast-enhanced images were multiple runs of 3D-coronal-T1W images, after injecting 10 cc of gadolinium-based contrast material (Dotarem™).

### Image interpretation

Two radiologists (NK and PL) assessed datasets independently. They were both blind to the clinical information of patients. Image interpretation was performed on a standard PACS workstation. They were first asked to assess the MDCTA images regarding the presence of artifacts and quality of contrast enhancement in the venous vasculature on a 5-point scale (5, no artifacts/excellent contrast; 4, few artifacts/good contrast; 3, moderate amount of artifacts/moderate contrast; 2, pronounced artifacts/poor contrast; 1, images uninterpretable because of artifacts/insufficient contrast). Subsequently they were asked to record whether the quality of images was sufficient for diagnostic evaluation^18^. They then assessed the MDCTA images regarding the presence/absence of CVT in the following six venous sinus segments: superior sagittal sinus, right transverse sinus, left transverse sinus, right sigmoid sinus, left sigmoid sinus and straight sinus. In cases of CVT, the presence of venous infarct, hemorrhage and other complications was recorded. In addition, the presence of an alternative diagnosis was documented. Finally, the diagnostic confidence about the presence or absence of a venous thrombosis was rated on a 5-point scale (5, absolutely certain; 4, very certain; 3, certain; 2, not very certain; 1, uncertain). ^18^ The readers could use multiplanar reconstruction (MPR) and intensity projection techniques at their convenience. They then tackled the discrepancies via discussion, and the consensus data are recorded here.

After having evaluated all MDCTA datasets, the readers evaluated the MRI + CE-MRV images as the reference standard in a separate session. In cases where two radiologists did not agree on the result of an MRI + MRV image, a third radiologist (BA), who was blind to the diagnosis of the first two, was asked to review the images. MRI images were evaluated in addition to CE-MRV images to increase the diagnostic accuracy. To eliminate recall bias, the MRI + CE-MRV images were interpreted at least 4 weeks after MDCTA dataset evaluation.

### Statistical analysis

All the obtained data were collected on a database. Sensitivity, specificity, and diagnostic accuracy of MDCTA for diagnosis of CVT were calculated, considering CE-MRV results as the gold standard. Data were analyzed using IBM SPSS version 22. A *p*-value of less than 0.05 was considered as statistically significant.

### Ethical considerations

The personal information of patients, including names, were removed from the images and was replaced with a code unique to every individual. The medical and personal information of patient were not shared outside the research group without their written consent. This study was approved by the ethics committee of Mashhad university of medical sciences (approval code: IR.MUMS.MEDICAL.REC.1398.470). The review board waived informed consent due to the observational nature of the research.

## Results

A total of 245 patients were found on our PACS database for whom brain CTA and contrast enhanced MRV were requested at the admission. The mean age of patients was 42.5 (± 15.2) years; in detail, the male maximum and minimum age were 86 and 18 years, respectively, while females were 81 and 17 years, respectively. There were 104 (42.4%) male and 141 (57.6%) female patients. None of the studied patients demonstrated any signs and symptoms of contrast-induced nephropathy such as skin rash, nausea, vomiting, or other clinical findings suggestive of the creatinine rising during the 48 h after imaging. The reference standard (MRI + CE-MRV) consensus reading identified CVT in 28 (11.4%) patients. Among the patients negative for CVT, meningioma was seen in 3, pituitary adenoma was found in one, and SAH without any underlying vascular abnormality was seen in 4 patients (alternative diagnoses). In patients with CVT, the most commonly involved dural vein segments were superior sagittal (57.1%) and right transverse (57.1%) sinuses, followed by left transverse sinus (50%). The less frequently involved segment was internal cerebral vein (10.7%). Cortical vein thrombosis was not evaluated in this study. Demographic data and thrombus location are summarized in Table 1. In some patients in whom CVT was not present at the time of evaluation, other intracranial pathologies were detected, among which, intracranial hemorrhage was the most common finding. In 122 patients, the readers found diagnoses other than CVT. The most frequent alternative diagnosis was arterial infarct that was seen in 36 (14.9%) of cases. Non-CVT pathologies detected in the MRI images of patients are summarized in Table 2.

**Table 1:** Demographics, and the frequency of CVT-related findings in 245 evaluated patients.

	Overall	CVT	No-CVT	P value
Number (%)	245	28 (11.4)	217 (88.6)	
Sex (male/female)	104/141	14/14	89/126	0.25
Age (SD)	42.5 (15.2)	41.5 (13.4)	42.6 (15.5)	0.3
**Location of thrombosis, number (%)**				
Superior sagittal	16 (6.4)	16 (57.1)	–	–
Left transverse	14 (5.7)	14 (50)	–	–
Right transverse	16 (6.4)	16 (57.1)	–	–
Left sigmoid	9 (3.7)	9 (32.1)	–	–
Right sigmoid	11 (4.3)	11 (39.3)	–	–
Straight	7 (2.8)	7 (25)	–	–
Galen vein	6 (2.4)	6 (21.4)	–	–
Internal cerebral vein	3 (1.2)	3 (10.7)	–	–
Venous infarct	15 (6)	15 (53.6)	–	–

**Table 2:** The frequency of other pathologies detected in the MRI images of 245 patients evaluated for CVT.

Intracranial hemorrhage	47 (19.2)
Arterial aneurysm	2 (0.8)
Subarachnoid hemorrhage	9 (3.7)
Arterial infarct	33 (14.6)
Arteriovenous malformation	6 (2.4)
Cavernous angioma	2 (0.8)
Carotid-cavernous fistula	1 (0.4)
Demyelinating disease	2 (0.8)
Dural AVF	1 (0.4)
Deep vein anomaly	1 (0.4)
Encephalitis	1 (0.4)
Intracranial tumor	5 (2)
Subdural hemorrhage	6 (2.4)

*AVF* arteriovenous fistula.

The overall brain CTA quality was interpreted as sufficient in 242 (98.8%) of cases. Only three (1.2%) cases were evaluated as uninterpretable, and were excluded from further analysis. The presence of artifacts was generally reported as no artifact or few artifacts (mean score of 4.8 for reader 1, and 4.6 for reader 2). The vessel contrast was generally assessed as excellent (mean score of 4.9 for reader 1 and 4.6 for reader 2). The mean diagnostic confidence about the presence/absence of acute CVT was 4.6 (very certain).

In the blinded MDCTA evaluation, there was a sensitivity of 96.1%, specificity of 98.6% and accuracy of 98.3% in detecting CVT. There were one case of false negative and three cases of false positive CTA readings in our series. In all false positive cases, the false impressions of thrombosis were in transverse sinuses. In the one case of false negative reading, thrombosis was located in the hypoplastic left transverse and sigmoid sinuses.

## Discussion

**Figure 1:**
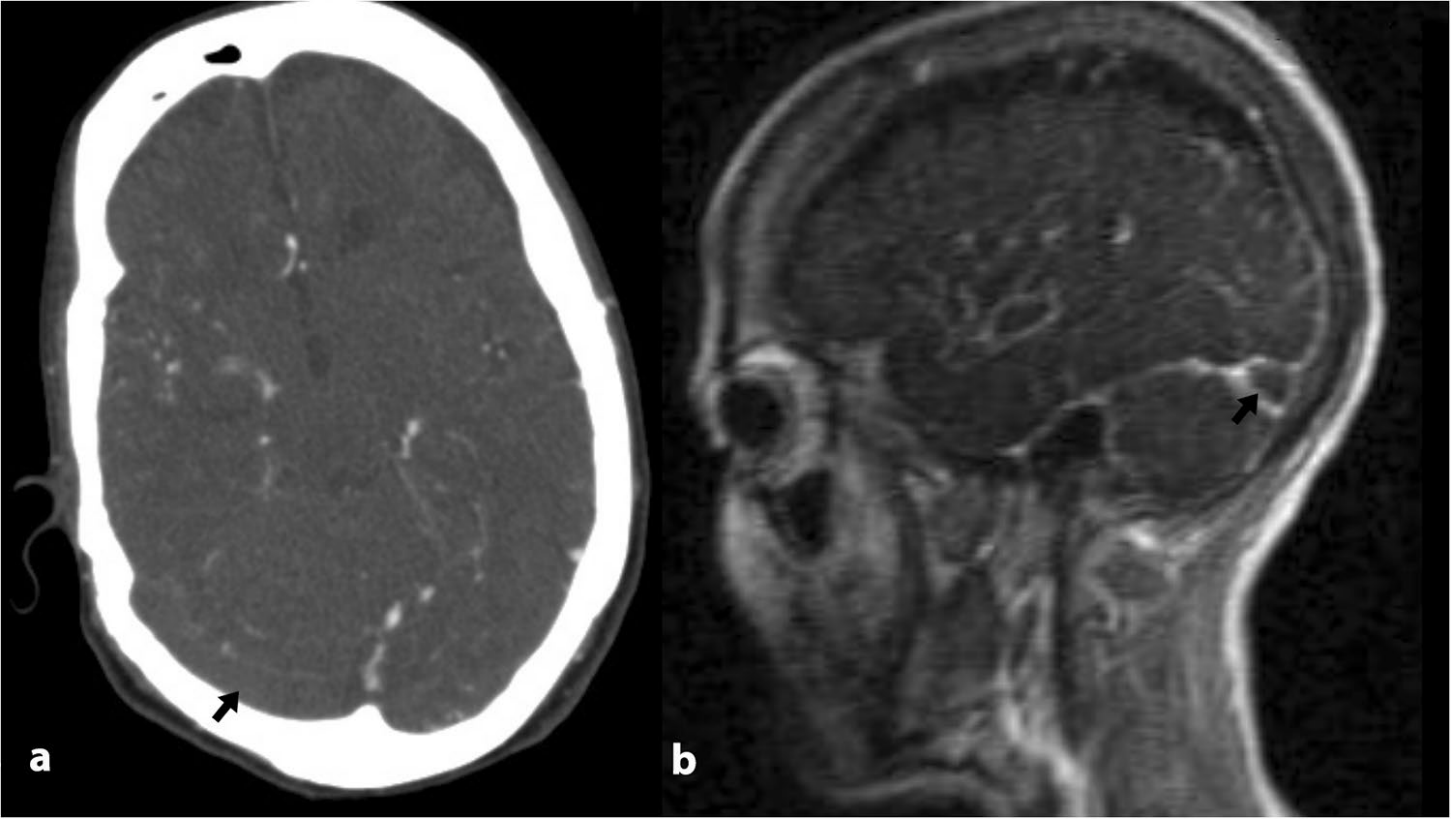
(**a**) Axial brain CT angiography shows filling defect in the right transverse sinus (black arrow). (**b**) Sagittal contrast enhanced brain MR venography shows filling defect in the right transverse sinus (white arrow).

Cerebral venous thrombosis is a disorder with a challenging diagnosis, mostly due to wide variety of clinical manifestations^19^. Although digital subtraction angiography is considered the gold standard diagnostic measure, it is now rarely acquired and contrast-enhanced MRV is considered the standard diagnostic procedure and the non-invasive gold standard. Contrast-enhanced MRV is associated with limitations like lack of availability in many medical centers, being expensive and time-consuming, being prone to motion artifacts, and limited use in patients with cardiac pace makers and claustrophobia. Gadolinium-based contrast materials used in MRV are also contraindicated in certain conditions like pregnancy and end-stage renal disease. As CT scan is still the initial neuroimaging test in patients presenting with new-onset neurologic symptoms such as headache, seizure, mental alteration, or focal neurological signs and is readily available in most healthcare centers, it is tempting to define a standard diagnostic protocol for CVT using computed tomography^20^. Non contrast brain CT scan is often normal, but may show findings like increased attenuation within the affected dural sinuses^21^. However this finding is insensitive and is reported in only 30% of cases of acute CVT^20^. Brain CT venography (CTV) has been reported to be a promising technique in evaluating CVT^19^. Small studies comparing multiplanar CT/CTV vs. DSA showed 95% sensitivity and 91% specificity^20^. European Academy of Neurology suggested that CTV can be used as a reliable alternative to both DSA and MRV for the diagnosis of CVT in patients with suspected CVT, but the quality of evidence is stated to be very low^22^. Due to the insidious nature of presentations in CVT, brain MDCTA might be acquired in some patients to rule out arterial pathologies like aneurysms, and performing an additional brain CTV will duplicate the radiation exposure. As the arterial and venous phases are too close in cerebral circulation and dural sinuses are generally visible in brain MDCTA images^17^, we postulated that brain MDCTA could be a promising diagnostic technique that could replace both contrast-enhanced brain MRV and brain CTV. Our results demonstrated a sensitivity of 96.1% and specificity of 98.6% for brain MDCTA in diagnosing acute CVT. Other previous studies also suggested great diagnostic accuracy for MDCTA^17^, but this is the first study that evaluated this blindly on a large sample size collected from a 5-year data archive. The results showed 96.1% sensitivity and 98.6% specificity for MDCTA in diagnosing acute CVT using contrast enhanced MRV as the reference. Both readers found the images of excellent quality regarding motion artifact and vessel contrast, and reported a high diagnostic confidence (4.6/5). This is mostly because the new-generation multi-detector scanners have high spatial and high temporal resolution, enabling high quality 3D and multiplanar reconstruction and detailed examination of all sinus segments (Fig. 1).

**Figure 2:**
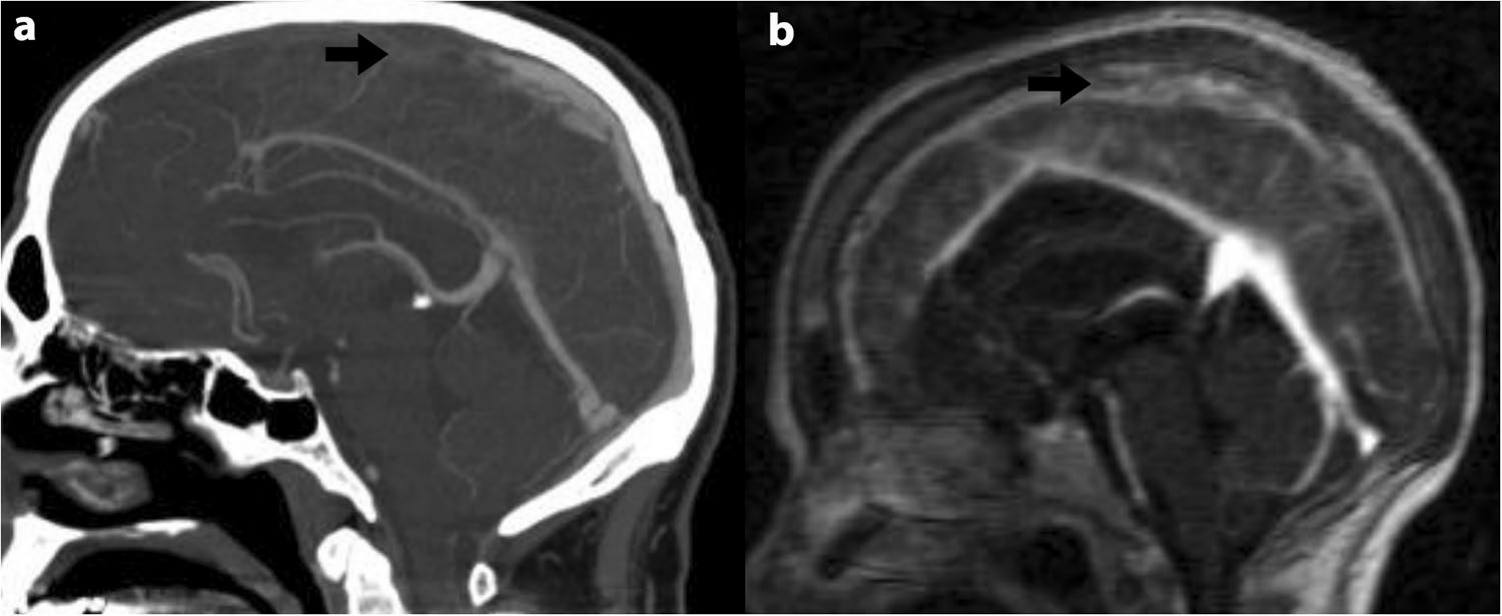
False negative CTA reading in a 38 y/o woman with CVT. The Sagittal reconstruction CTA (**a**) shows an irregular filling defect within the superior sagittal sinus (black arrow) that was missed by the readers. The filling defect (white arrow) is also seen in the sagittal MR venography.

In the one case of false negative results of MDCTA reading, the patient had a partial filling defect in the superior sagittal sinus, and there was discrepancy among the readers, even while interpreting the MRV images (Fig. 2). The presence of venous infarct led to the final decision of the presence of CVT in the patient. In all three cases of false positive results that were all attributed to the transverse and sigmoid sinuses, slow flow was detected in the same sinus segments on the MRI images of the patients.

It should be mentioned that brain MDCTA is associated with some drawbacks including radiation concerns, potential for iodine contrast material allergy, and issues regarding the use of contrast in the setting of impaired renal function^17^. In detail, despite studies that have stated that contrast-induced nephropathy is a rare event in MDCTA, conditions such as previous chronic renal insufficiency and diabetes were associated with the contrast-induced nephropathy in patients undergoing MDCTA^23^.

The study has some limitations, one of them being its retrospective nature. Also, the maximum time interval of seven days between CE-MRV and MDCTA could have been even less, resulting in a more accurate comparison between the two modalities. Cortical vein thrombosis is not evaluated in this study. Cortical vein thrombosis is usually associated with deep vein thrombosis, and its presence does not change the treatment plan. Isolated cortical vein thrombosis is a rare entity for which diagnosis needs a T2* sequence. This sequence is not routinely obtained. As we could not confirm the presence of cortical vein thrombosis on the MRI images, we did not evaluate it in the CTA images.

## Conclusion

According to the lethal outcomes of the CSVT, exploring a highly sensitive and specific diagnostic method that is more available and faster than gold standards such as digital subtraction angiography and CE-MRV, seems necessary to reduce this rare event. Based on the present study, in emergency settings, and in centers in which MRI scanners are not available, MDCTA can be used instead of CE-MRV for the diagnosis of CSVT with good sensitivity and specificity. Further investigations are required to determine the advantages and disadvantages of the MDCTA in detecting CSVT.

## Data Availability

The datasets used and/or analyzed during the current study is available from the corresponding author on reasonable request.
